# New therapeutic strategies in neuroblastoma: combined targeting of a novel tyrosine kinase inhibitor and liposomal siRNAs against *ALK*

**DOI:** 10.18632/oncotarget.4342

**Published:** 2015-06-20

**Authors:** Daniela Di Paolo, D. Yang, Fabio Pastorino, Laura Emionite, Michele Cilli, Antonio Daga, Elisa Destefanis, Annarita Di Fiore, Francesca Piaggio, Chiara Brignole, Xiaobao Xu, Chris Liang, James Gibbons, Mirco Ponzoni, Patrizia Perri

**Affiliations:** ^1^ Laboratorio di Oncologia, Istituto G. Gaslini, Genoa, Italy; ^2^ Sundia MediTech Company, Ltd., Shangai, China; ^3^ Animal Facility, IRCCS Azienda Ospedaliera Universitaria San Martino-IST Istituto Nazionale per la Ricerca sul Cancro, Genoa, Italy; ^4^ Laboratorio Trasferimento Genico, IRCCS Azienda Ospedaliera Universitaria San Martino-IST Istituto Nazionale per la Ricerca sul Cancro, Genoa, Italy; ^5^ Xcovery LLC, West Palm Beach, FL, USA; ^6^ Present address: Centre for Inherited Cardiovascular, IRCCS Politecnico San Matteo, Pavia, Italy

**Keywords:** neuroblastoma, ALK-inhibitor, X-396, RNA-interference, targeted nanoliposomes

## Abstract

Many different aberrations in the Anaplastic Lymphoma Kinase (*ALK*) were found to be oncogenic drivers in several cancers including neuroblastoma (NB), therefore ALK is now considered a critical player in NB oncogenesis and a promising therapeutic target. The ALK-inhibitor crizotinib has a limited activity against the various *ALK* mutations identified in NB patients.

We tested: the activity of the novel ALK-inhibitor X-396 administered alone or in combination with Targeted Liposomes carrying *ALK*-siRNAs (TL[*ALK*-siRNA]) that are active irrespective of ALK gene mutational status; the pharmacokinetic profiles and the biodistribution of X-396; the efficacy of X-396 *versus* crizotinib treatment in NB xenografts; whether the combination of X-396 with the TL[*ALK*-siRNA] could promote long-term survival in NB mouse models.

X-396 revealed good bioavailability, moderate half-life, high mean plasma and tumor concentrations. X-396 was more effective than crizotinib in inhibiting *in vitro* cell proliferation of NB cells and in reducing tumor volume in subcutaneous NB models in a dose-dependent manner.

In orthotopic NB xenografts, X-396 significantly increased life span independently of the *ALK* mutation status. In combination studies, all effects were significantly improved in the mice treated with TL[*ALK*-siRNA] and X-396 compared to mice receiving the single agents.

Our findings provide a rational basis to design innovative molecular-based treatment combinations for clinical application in *ALK*-driven NB tumors.

## INTRODUCTION

Neuroblastoma (NB) is an embryonic tumor of the sympathetic nervous system, and is the most commonly solid malignancy diagnosed in the first year of life, accounting for about 9–10% of paediatric cancer mortality. NB is characterized by broad clinical phenotypes from spontaneous regression to fatal outcome in advanced stages with metastatic disease. Despite aggressive and multi-modal therapies (*i.e*. aggressive chemotherapy, surgery, radiation therapy, stem cell transplantation, immunotherapy) the effective treatment of advanced NB is still a challenge for paediatric oncology [1, 2].

Over the years, many genes have been encountered to have potential roles in NB pathogenesis, but the NB causing genes remained unidentified. The identification of germline and somatic activating mutations in the tyrosine kinase domain of the Anaplastic Lymphoma Kinase (ALK) gene as well as amplification, rearrangments and/or over expression of either mutated or wild-type ALK alleles-revealed that such aberrations are oncogenic drivers in NB and correlate with worse patients' outcome or unfavourable aggressive NB phenotype [3–7]. Notably, as both ALK and MYCN oncogene are located in close proximity to each other on chromosome 2p, amplification of MYCN can also involve amplification of the ALK locus [3, 4, 7, 8]. When expressed together, ALK and MYCN promote NIH3T3 cell transformation and both wild-type and activated mutant forms of ALK stimulate transcription of MYCN [9].

Therefore, ALK has emerged as a critical player in NB and tractable ‘oncogene’ opening new perspectives into the design and development of gene-targeted therapies for NB and other ALK-driven cancers [8, 10, 11], such as Non-Small-Cell Lung Cancers (NSCLC) [12] and Anaplastic Large-Cell Lymphomas (ALCL) [13] characterized by oncogenic ALK fusion proteins arising from chromosomal translocations.

ALK tyrosine kinase domain mutations account for 8–10% of diagnosed NB at three hot spots accounting for 85% of mutations: R1275 (43%), F1174 (30%), and F1245 (12%) and 13 minor sites, and correlated significantly with poorer survival in high- and intermediate-risk NB [3–8].

The success of various tyrosine kinase inhibitors in the treatment of different cancers as well as the increased number of human malignancies involving aberrant ALK activity, both in children and adults, encouraged the search and motivated the development of ALK-selective small-molecule inhibitors. Crizotinib, the only ALK-inhibitor approved by Food and Drug Administration (FDA, USA), showed limited activity against ALK-driven NB [14]. The mutated variants also showed differential *in vitro* crizotinib sensitivities [8].

The most aggressive *ALK*-mutation in NB, *ALK*^F1174L^, has been described as a relapse-specific mutation correlating with unresponsiveness to therapy [15]. *ALK*^F1174L^ also arises as a secondary event after an initial response to crizotinib [16] and is able to potentiate the oncogene activity of *MYCN* [17]. To overcome acquired resistance to crizotinib, different categories of next-generation ALK-inhibitors have been developed and are subject of various pre-clinical studies or early-phase trials [18]. However, there is no certainty that these compounds will be more potent than crizotinib against *ALK*-mutants. In this view, Xcovery (West Palm Beach, FL) has developed a novel and powerful small molecule ALK-inhibitor named X-396 (http://www.xcovery.com). The potency and the selectivity of X-396 were validated by comparison with other ALK-inhibitors available and tested in animal models of Non-Small-Cell Lung Cancer (NSCLC). Noteworthy, X-396 is active against multiple *ALK* variants founds in NSCLC, including *ALK* mutations associated with acquired resistance to crizotinib [10]. Indeed, in June 2012, X-396 entered a Phase 1 safety trial in patients with solid tumors [11] (for details see NCT01625234 at http://www.clinicaltrials.gov). Preliminary clinical data have shown that X-396 is generally well-tolerated and has anti-tumor activity in patients with NSCLC bearing an ALK fusion protein [19].

Based on these findings, we hypothesize that X-396 could be more effective than crizotinib on NB cells bearing in either of the two more common *ALK*-mutations, *ALK*^F1174L^ or *ALK*^R1275Q^ [20]. In addition, we believe that novel combination strategies that increase the efficacy of ALK-inhibitors like X-396 without increasing toxicities in children are potentially relevant.

To this regard, we have successfully developed and validated by *in vitro* and *in vivo* studies a RNAi-mediated therapeutic approach to selectively knockdown *ALK* expression by using NB targeted nanoliposomes [21, 22]. Since our formulation is a safe and powerful siRNA-based therapeutic tool for NB, we thought it may be ideal to combine with an ALK kinase inhibitor. Here we present results aimed at testing whether a combined therapeutic approach using the novel inhibitor X-396 working on ALK at protein level, and the NB targeted liposomal siRNAs against *ALK* working at mRNA level, could represent an improved strategy with additive and/or synergistic effects to promote long-term survival in NB xenografts.

## RESULTS

### X-396 is a kinase inhibitor with higher potency against *ALK*-mutated neuroblastoma-cell lines than crizotinib *in vitro*

Pre-clinical studies have shown that NB cell lines harbouring the F1174L mutation, the second most common *ALK*-mutation seen in NB tumors, are significantly more resistant to crizotinib than those harbouring the most common mutation, R1275Q [16, 20]. For this reason, we first measured ^3^H-thymidine uptake to assess the dose-dependent effect of X-396 and crizotinib on the growth of two NB cell lines, each one harbouring one of the hot spot-activating *ALK*-mutations: LAN-5 carrying R1275Q *ALK* mutation (*ALK*^R1275Q^) and SH-SY5Y carrying F1174L *ALK* mutation (*ALK*^F1174L^).

**Figure 1:**
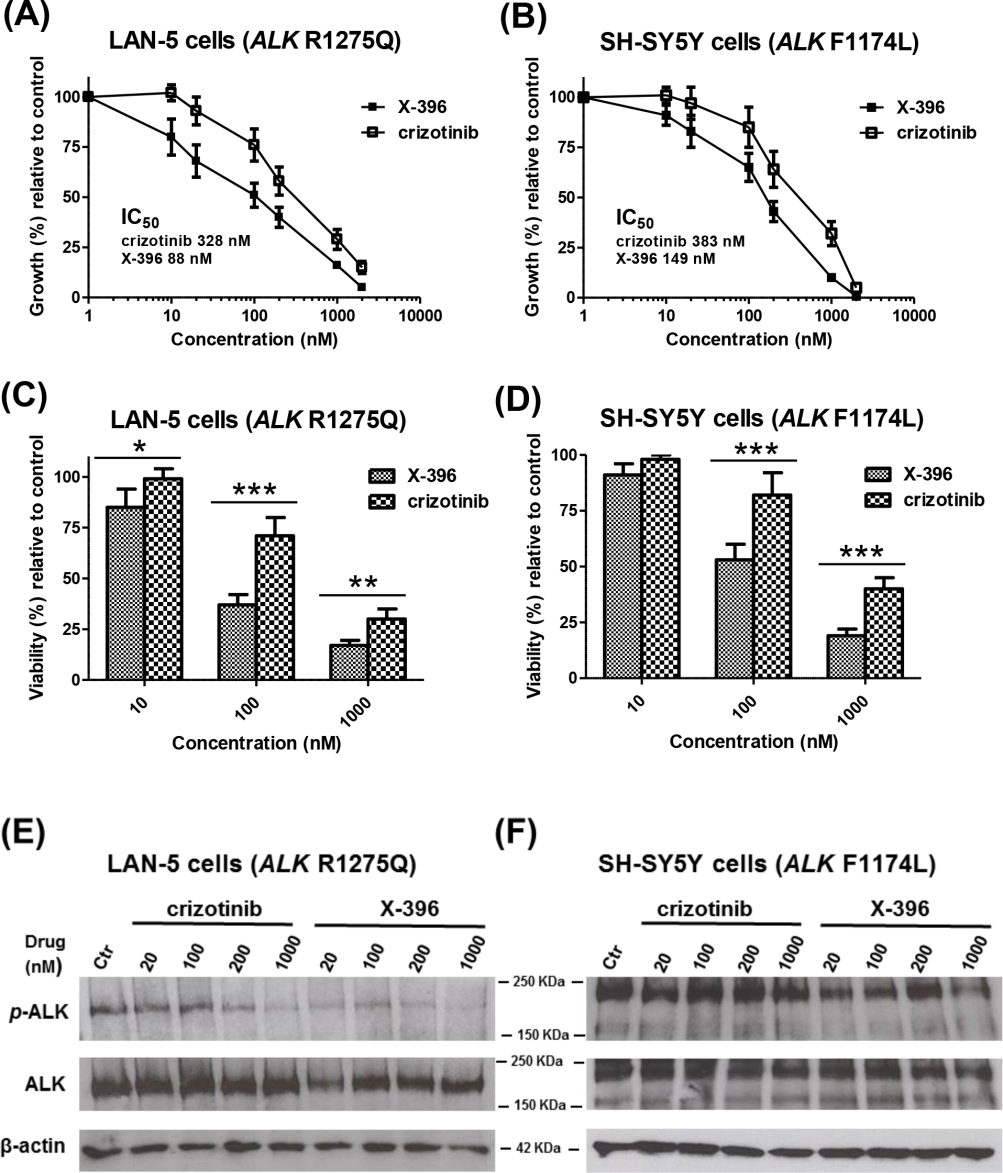
X-396 and crizotinib decrease the growth, viability and ALK-phosphorylation of LAN-5 and SH-SY5Y Neuroblastoma cell lines *in vitro* LAN-5 **A.** and SH-SY5Y **B.** cells were seeded in 96-well plates. The day after, cells were treated with various concentrations (1–2000 nM) of crizotinib or X-396. Results, derived from three different experiments, are expressed as mean percentage of ^3^H-thymidine incorporation 72 hours after initiation of treatment as compared to that of control cells (culture medium containing 0.01% dimethylsulfoxide). Error bars represent 95% confidence interval. Inset, indicate the concentration of drugs causing 50% inhibition of cell proliferation (IC_50_) for each cell line, evaluated by nonlinear regression (curve fit). LAN-5 **C.** and SH-SY5Y **D.** cells viability was measured by AlamarBlue staining 72 hours after treatment with 10–100–1000 nM of X-396 or crizotinib. Bars represent the cell viability, derived from three different experiments, as mean percentage of sextuplicate wells, considering the control level of cells (treated as in panels A and B) to be 100%. Error bars represent 95% confidence interval. *P* value (two-tailed) were calculated using the Student's *t* test with Welch's correction. *P < 0.05, **P < 0.01, ***P < 0.001. LAN-5 **E.** and SH-SY5Y **F.** cells were treated with various concentrations (20–1000 nM) of crizotinib or X-396 or 0.01% DMSO (Ctr) for 72 hours. Lysates were subjected to immunoblotting with the specific antibodies.

Although the slope of the curves between X-396 and crizotinib are similar, the differences between the elevations were significantly different in both cell lines. Specifically, in LAN-5 cells X-396 was 3.7-fold more potent than crizotinib (IC_50_: crizotinib 328 nM, X-396 88 nM) to decease cell growth (Figure 1A). Lower effect (total fold increase 2.57) was registered in SH-SY5Y cells(IC_50_: crizotinib 383 nM, X-396 149 nM) (Figure 1B).

We next examined the activity of X-396 on the cell viability of cultured NB cells by AlamarBlue staining. Treatment with X-396 induced a statistically significant dose-dependent decrease in cell viability compared with the same dose of crizotinib (Figure 1C, 1D).

To confirm the target specificity of X-396, we assessed the ability of the compound to reduce the endogenous ALK phosphorylation in SH-SY5Y and LAN-5 NB cells. Compared to crizotinib, X-396 inhibited ALK phosphorylation at lower concentrations of drug (Figure 1E, 1F and Supplementary Figure S1).

The above results indicated that X-396 is an *ALK*-inhibitor more powerful and gene-specific than crizotinib in accordance with previous findings obtained by Lovly et al [10].

### Pharmacokinetic profile, biodistribution properties and anti-tumor activity of X-396 in subcutaneous NB mouse model

**Figure 2:**
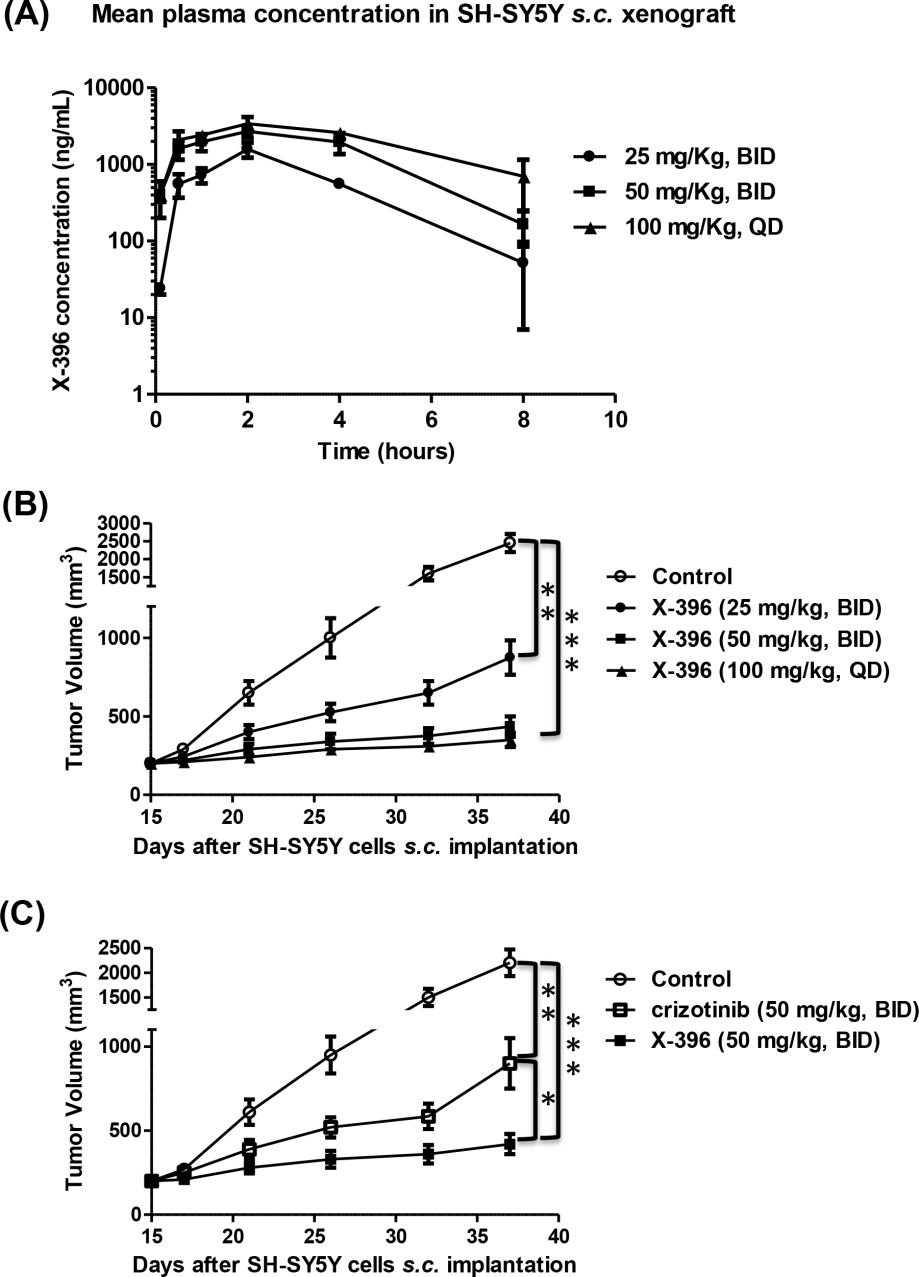
Pharmacokinetic profiles and tumor volume measurement over time after multiple *in vivo* administration of X-396 **A, B, C.** SH-SY5Y NB cells were xenografted in Balb/c *nude* mice and randomly divided in groups. Mice were treated by oral gavage (OG) with X-396 following different schedules: 25 mg/Kg *bis in die* (BID), 50 mg/kg BID and 100 mg/kg *quaque die* (QD) (A, B). At different time points blood sample were collected and X-396 concentration was measured (A). Results are expressed as mean plasma concentration of X-396 ± Standard Deviation (SD). (B) Tumors were measured at fixed times with a calliper, and volume calculated. Error bars ± SD. C) Comparison of X-396 and crizotinib administered at the same dose. NB-bearing mice were OG treated with 50 mg/kg BID of X-396 or crizotinib and tumor volume determinated over time. Error bars ± SD. The statistical significance of differential findings between experimental groups and controls was determined by one-way analysis of variance (ANOVA) with the Tukey's multiple comparison test. *P < 0.05, **P < 0.01, ***P < 0.001

We next investigated the effects of X-396 *in vivo*, in SH-SY5Y xenografts. Pharmacokinetic profiles revealed that X-396, after multiple oral administrations by gavage in NB xenograft mice, showed substantial bioavailability, moderate half-life and slow absorption *in vivo* (time of the maximum concentration (T_MAX_: 2 h). At the low dose of 25 mg/kg, the mean plasma concentration 2 hours after the last dosing was 1284 ng/mL or about 2.3 μM which is 15x that of the IC_50_ of inhibiting the SH-SY5Y cell growth, *in vitro* (Figure 2A and Table 1).

**Table 1:** The non-compartment pharmacokinetic parameters of X-396 after multiple oral gavage administrations (3 different doses for 14 days) in Balb/c nude mice with SH-SY5Y xenograft tumors

Parameter	Dosage (mg/Kg) and Frequency		
	25 (BID)	50 (BID)	100 (QD)
**AUC_(0-t)_ (ng/mL ×h)**	4976.9	12685.2	16847.9
**AUC_(0-∞)_ (ng/mL ×h)**	5069.9	13199.7	21288.5
**MRT_(0-t)_ (h)**	2.60	2.86	3.21
**V_z_/F (L/Kg)**	8.62	11.63	29.37
**CL_z_/F (L/h/Kg)**	4.93	3.79	4.70
**T_1/2_ (h)**	1.21	2.13	4.33
**T_max_ (h)**	2.00	2.00	2.00
**C_max_ (ng/mL)**	1581.1	2718.4	3230.4

Abbreviation: AUC = area under the plasma drug concentration; MRT = mean residence time; V_z_/F = volume of distribution; CL_z_/F = clearance; T_1/2_ = terminal half life; T_MAX_ = time of maximum concentration; C_MAX_ = maximum plasma concentration

The tissue concentration of X-396 was also examined in these mice. It clearly indicated that the X-396 concentrations were higher in the tumor site than in the plasma in each schedule of treatment performed, reaching 32 fold-ratio increase 8 h after the last treatment with 50 mg/kg BID (Table 2). Since the plasma and tumor concentrations of X-396 are well above the IC_50_ of inhibiting cell growth, we expected that X-396 should be able to inhibit tumor growth.

**Table 2:** Plasma, brain and tumor concentration of X-396 after oral gavage administrations

A						
Time (hr)		Plasma concentration (ng/mL)	Brain concentration (ng/g)	Tumor concentration (ng/g)	BPR	TPR
2	Mean SD	1283.97 151.94	69.75 14.47	2509.92 71.11	0.054	1.96
4	Mean SD	552.03 91.41	47.46 3.60	2085.72 483.89	0.09	3.78
8	Mean SD	52.15 45.37	12.26 4.68	929.84 364.16	0.34	17.83

B						
Time (hr)		Plasma concentration (ng/mL)	Brain concentration (ng/g)	Tumor concentration (ng/g)	BPR	TPR
2	Mean SD	2539.14 253.43	198.21 14.21	10469.07 1782.63	0.08	4.12
4	Mean SD	1960.29 657.59	195.98 67.77	11280.32 5454.42	0.1	5.76
8	Mean SD	165.39 88.08	34.62 13.02	5270.86 2186.48	0.21	31.87

C						
Time (hr)		Plasma concentration (ng/mL)	Brain concentration (ng/g)	Tumor concentration (ng/g)	BPR	TPR
2	Mean SD	2485.94 637.46	387.00 127.28	12045.60 1199.65	0.16	4.85
4	Mean SD	2563.48 328.99	328.32 80.98	14933.3 5099.85	0.13	5.83
8	Mean SD	704.86 670.10	138.64 129.61	11860.58 3491.69	0.2	16.83

**A.** (25 mg/kg, BID, for 14 days); **B.** (50 mg/kg, BID, for 14 days); **C.** (100 mg/kg, QD, for 14 days) in Balb/c nude mice with SH-SY5Y xenograft tumors. BPR = Brain Plasma Ratio, TPR = Tumor Plasma Ratio

Indeed, X-396 was able to significantly reduce the tumor volume in a subcutaneous SH-SY5Y NB model in a dose-dependent manner (Figure 2B) and its efficacy was more pronounced than that obtained with crizotinib when administered at the same dose (Figure 2C). These findings suggest that the same anti-tumor activity may be achieved by administration of lower doses of X-396 with respect to crizotinib, in keeping with results obtained in other animal models by Lovly et al [10].

The Maximum Tolerated Dose (MTD) was not identified with any given regimens and no signs of adverse effect were observed (*i.e*. dehydration, severe weight loss, Supplementary Figure S2) during the treatment.

### *In vivo* anti-tumor activity of X-396 against human NB orthotopic xenografts

We next asked whether the above anti-tumor results could be recapitulated in a more clinically relevant mouse model. To this purpose, we explored the effects of X-396 in biologically relevant orthotopic mouse models [23], obtained by implanting of Luciferase-stably-transduced NB cells, SH-SY5Y-Luc and LAN-5-Luc, into the adrenal gland of *nu/nu* mice.

To avoid the possible stressful mice conditions, due to the repeated *oral gavage* in the same day, BID, we decided to administrate 50 mg/kg and 100 mg/kg of X-396 in two mice groups only once a day (QD), starting 7 days post cell implantation.

**Figure 3:**
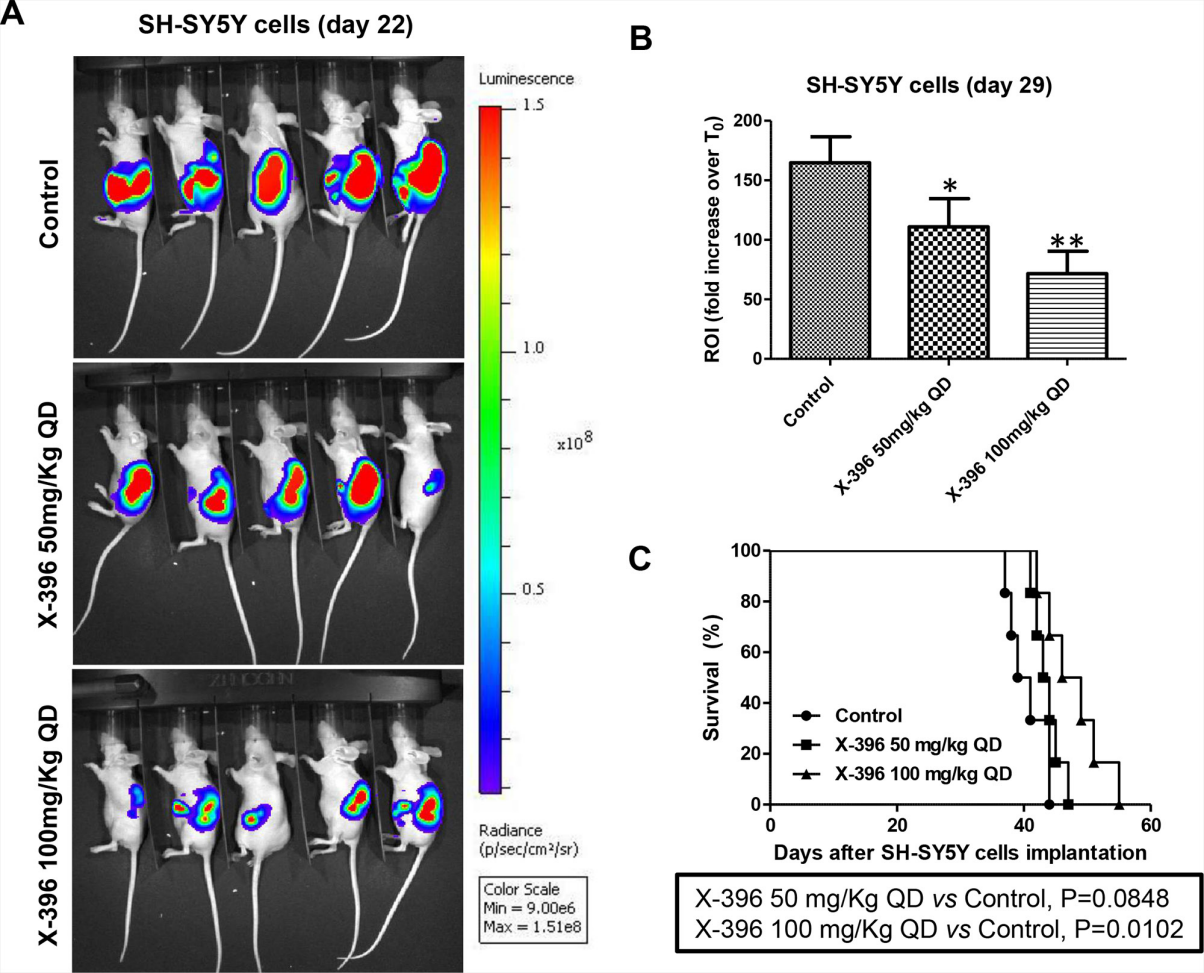
Dose-dependent efficacy of X-396 treatment in SH-SY5Y cells orthotopic-bearing mice SH-SY5Y-Luc cells were implanted orthotopically into the left adrenal gland of immunodeficient mice. NB-bearing mice were randomly assigned to three groups and treated 7 days after the cells transplantation (considered T_0_) *via* OG QD with X-396 at the indicated doses or vehicle alone (Control) for 21 days. Body weight and general physical status were recorded daily, and mice were sacrificed by cervical dislocation after the administration of xilezine when they showed signs of poor health. **A.** Lateral (cell implantation side) images of BLI intensity from five NB-bearing mice implanted with SH-SY5Y-Luc cells evaluated at day 22. The relative levels of bioluminescence are shown as a pseudocolor display, with red and violet representing the strongest and weakest photon fluxes, respectively. **B.** Quantification of the *in vivo* Luciferase signal of the tumor area expressed as fold increase of photon counts over time in the region of interest (ROI) computed at time 29 day respect to T_0_. Error bars represent 95% confidence intervals. *P* value (two-tailed) were calculated using ANOVA with the Tukey's multiple comparison test. *P < 0.05, **P < 0.01. **C.** Survival curves of *nu/nu* NB-bearing mice (*n* = 6 for each group) in response to treatments. Kaplan-Meyer method & Log Rank (Mantel-Cox) test was applied to build up and compare the survival curves between analyzed groups.

**Figure 4:**
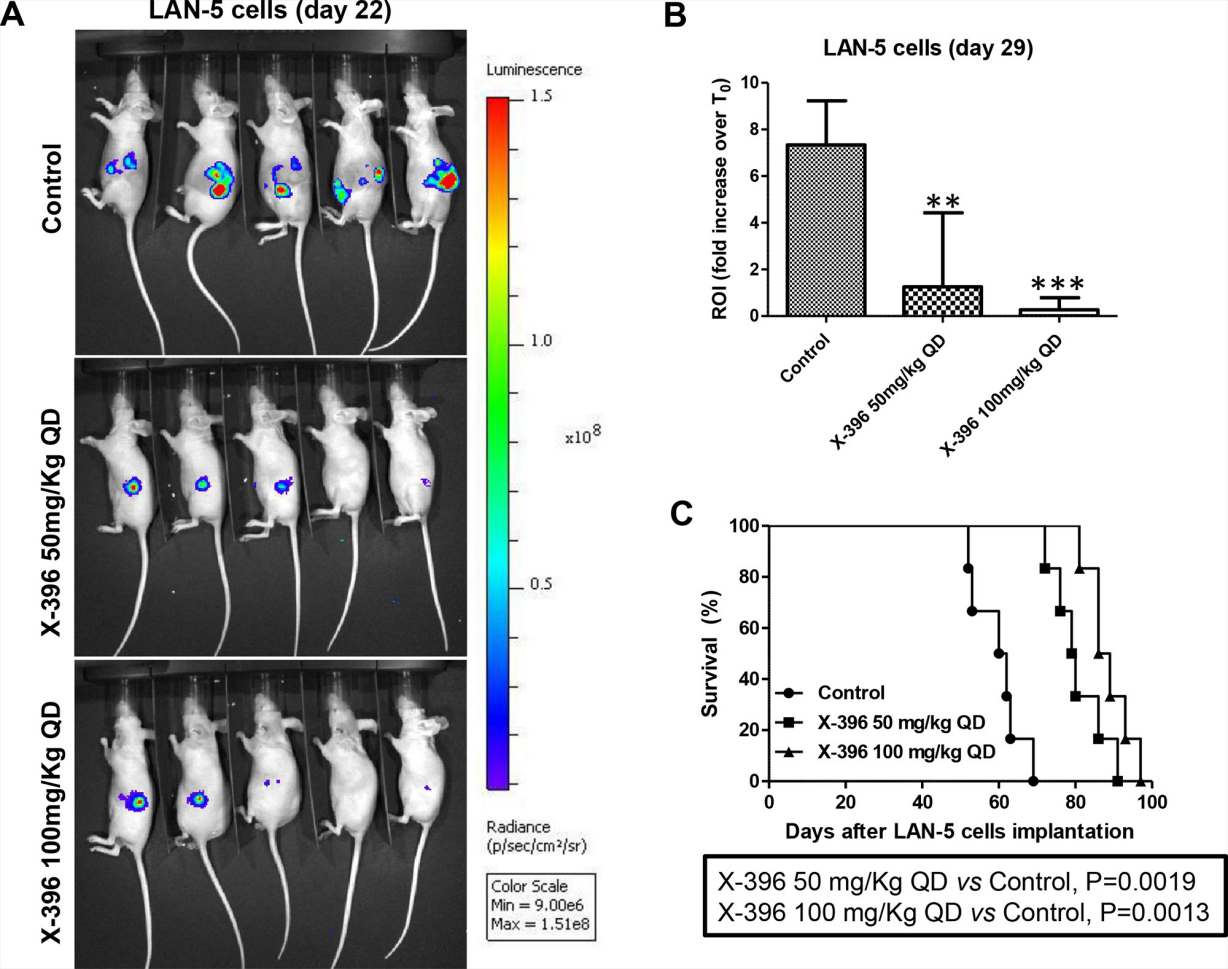
Dose-dependent efficacy of X-396 treatment in LAN-5 cells orthotopic-bearing mice Schedule of treatments as in Figure 3 **A.** Lateral (cell implantation side) images of BLI intensity from five NB-bearing mice implanted with LAN-5-Luc cells evaluated at day 22. **B.** Quantification of the ROI as above. **P < 0.01, ***P < 0.001. **C. S**urvival curves of NB-bearing mice (*n* = 6 for each group) in response to treatments. Kaplan-Meyer method & Log Rank (Mantel-Cox) test was applied.

Treatments with X-396 did not revealed any sign of toxicities (*i.e*. severe weight loss, dehydration or abdominal dilatation). As clearly shown by *in vivo* BLI (Figure 3A and 4A) capturing the Luciferase signal intensity at day 22, X-396 treatments were able to slow down the primary tumor growth. All data were confirmed and quantified at day 29 by the fold increase of photon counts over the time in the tumor region (Figure 3B and 4B), indicating a significant dose-dependent anti-tumor activity of X-396 in both mouse models. In SH-SY5Y xenografts the lower dose of X-396 (50 mg/kg QD) did not significantly increase the mice life span compared to control group, while a significant improvement of the anti-tumor effect was obtained with the higher dose (100 mg/kg QD) (Figure 3C). This is consistent with the high brain tissue concentration of X-396 when dosed at 100 mg/kg, where the mean is 138 ng/g 8 hours after dosing. This concentration (∼246 nM) is 1.7x the IC_50_, while that at 50 mg/kg is only 34.6 ng/mL or ∼61 nM which is below the IC_50_ of 149 nM (Table 2). Noteworthy, either 50 mg/kg QD or 100 mg/kg QD doses of X-396 exerted a significant increased life span in LAN-5-bearing mice (Figure 4C).

### Combined anti-tumor activity of X-396 and NB-targeted nanoparticles entrapping siRNA against orthotopic and pseudometastatic human NB xenografts

To determine whether the silencing of *ALK*, independently of its mutational status, could improve the anti-tumor efficacy of the *ALK*-inhibitor X-396, we decided to introduce our well established NB-targeted nanoparticles entrapping siRNA for specific *ALK*-knockdown [21, 22] in the treatment schedule.

**Figure 5:**
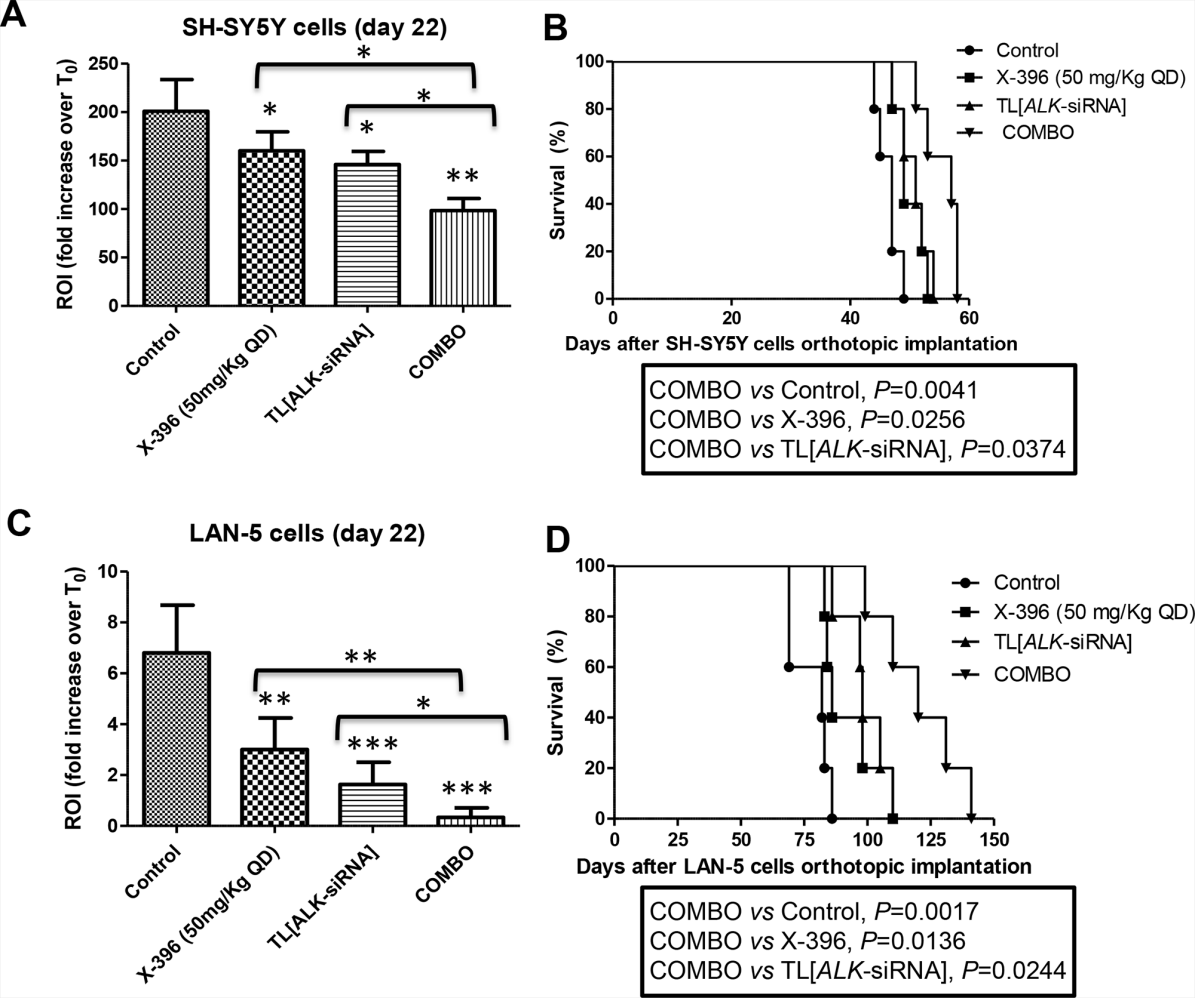
Targeted Liposomes entrapping siRNA against ALK show therapeutic efficacy in combination with X-396 in NB orthotopic models SH-SY5Y-Luc **A, B.** and LAN-5-Luc **C, D.** NB-tumor cells were orthotopically implanted in *nu/nu* mice. 7 days after the cells implantation, mice were treated for 21 days with 50 mg/kg QD of X-396 (*via* OG) and/or intravenously (*i.v*.) with 1 mg/Kg (20 μg/injection per mouse) twice a week for 3 weeks of Targeted Liposomes (TL) entrapped siRNA against *ALK* (TL[*ALK-*siRNA]), each administered individually and in combination (COMBO). A, C) Quantification of fold increase of the *in vivo* Luciferase signal of the lateral region of interest computed at time 22 days respect to T_0_ (starting day of the treatment). Error bars represent 95% confidence intervals. *P* value (two-tailed) were calculated using ANOVA with the Tukey's multiple comparison test. *P < 0.05, **P < 0.01, ***P < 0.001; B, D) The therapeutic effects of treatments were evaluated in terms of overall survival of NB-bearing mice (*n* = 5 for each group). Kaplan-Meyer method & Log Rank (Mantel-Cox) test was applied.

Seven days after orthotopic implantation of SH-SY5Y-Luc and LAN-5-Luc-transduced cells, mice were treated for 21 days with 50 mg/kg QD of X-396 or with 1 mg/Kg of targeted liposomes entrapped siRNA against *ALK* (TL[*ALK-*siRNA]) administered either individually or in combination (COMBO). As clearly summarized by the ROI values computed at day 22 with respect to the control group at T_0_, TL[ALK-siRNA] determined a statistically significant decrease of the signal in both cell lines (Figure 5A and 5C). Noteworthy, the combination of X-396 *plus* TL[*ALK-*siRNA] treatment led to further significant reduction of ROI signals respect to the single agents. These results were supported by a significant increase of the mice life span, as showed in Figure 5B and 5D.

**Figure 6:**
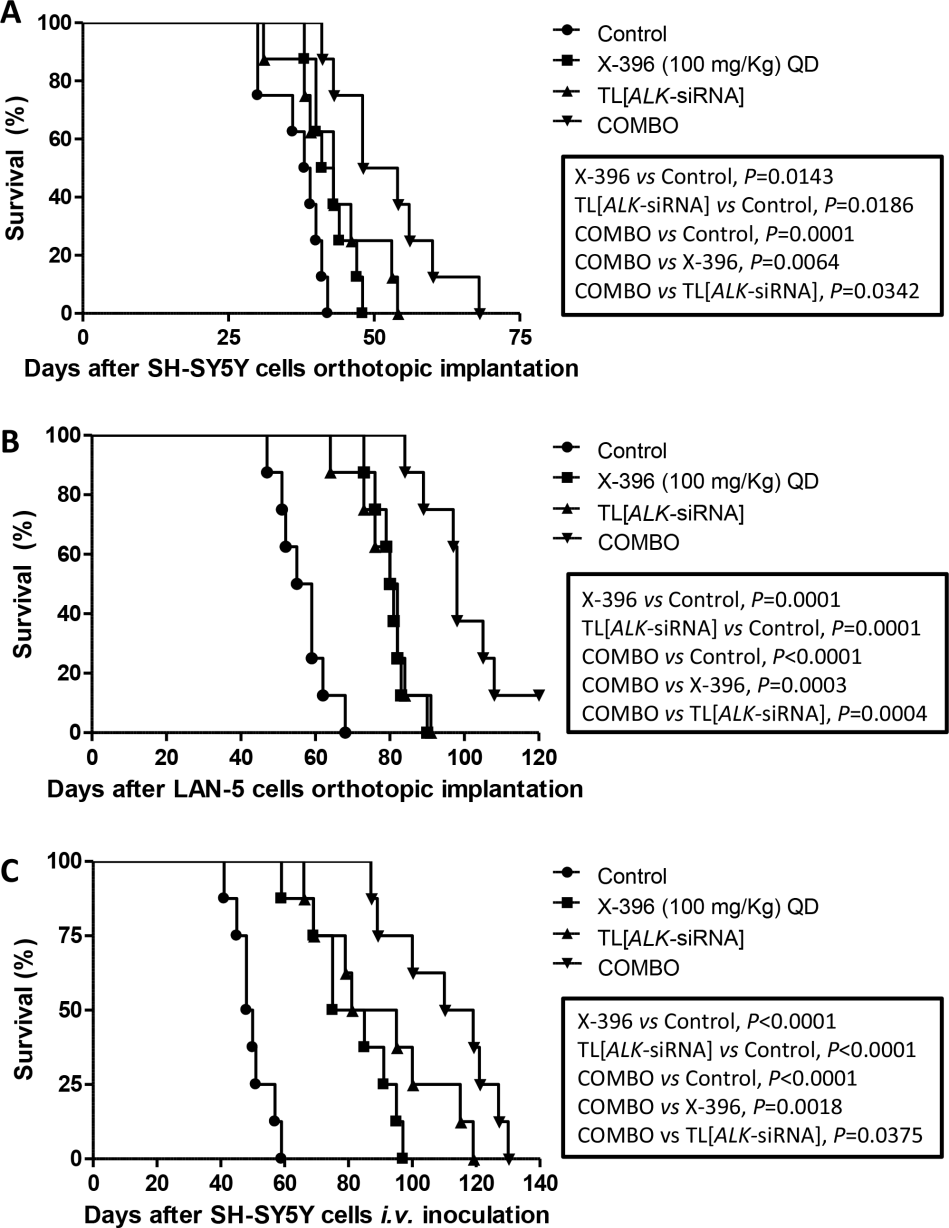
Enhanced tumor growth inhibition *in vivo* by TL[ALK-siRNA] plus X-396 in orthotopic and pseudometastatic NB models SH-SY5Y **A.** and LAN-5 **B.** NB tumor cells were orthotopically implanted in *nu/nu* mice (*n* = 8 for each group).7 days after the cells implantation, mice were treated for 21 days with 100 mg/kg QD of X-396 (*via* OG) and/or *i.v.* with 1 mg/Kg (20 μg/injection per mouse) twice a week for 3 weeks of TL[*ALK-*siRNA], each administered individually or in combination (COMBO). **C.** For the pseudometastatic model, *nu/nu* mice were injected *i.v*. in the tail vein with SH-SY5Y NB cells. Mice (*n* = 8 for each group) were randomly assigned to four groups and treated 4 hours after the cells inoculation as above. The therapeutic efficacy of the combined treatment respect to single treatments and to control group (vehicle alone) was evaluated in terms of overall survival. Kaplan-Meyer method & Log Rank (Mantel-Cox) test was applied.

Based on these encouraging results, we decided to verify whether the increase of X-396 dose to 100 mg/Kg QD was able to further ameliorate tumor growth in the mice. As shown in Figure 6, X-396 and TL[*ALK-*siRNA] used alone induced a statistically significant increase of mice life span with respect to control mice in both orthotopic (panels A, B) and pseudometastatic experimental models, being the latter able to mimic the Minimal Residual Disease [24] (panel C). The median survival of mice treated with X-396 *versus* controls was 42 *vs* 38.5 (panel A), 80.5 *vs* 57 (panel B) and 80 *vs* 49 (panel C) days. Regarding the TL[*ALK-*siRNA] groups, the median survival was 43 *vs* 38.5 (panel A), 81 *vs* 57 (panel B) and 88 *vs* 49 (panel C) days.

Noteworthy, the combination of X-396 plus TL[*ALK-*siRNA] significantly enhanced the anti-tumor activity of the single agents (Figure 6A, 6B, 6C). The combined treatment revealed values of median survival equal to 51 and 98 days for SH-SY5Y and LAN-5 orthotopic implant, respectively, and 114.5 days for the pseudometastic model.

## DISCUSSION

Despite intensive multimodal treatments (*i.e*. combination of isotretinoin, myeloablative therapy with stem-cell rescue and immunotherapy), the outcome of high-risk NB-patients remain very poor and the 2-years progression-free survival was improved only by 20% [25]. Since a considerable cohort of children are refractory to conventional approaches and/or can face relapse [26], new improved treatment options are needed.

Many studies on familial and sporadic cases with advanced NB have shown either germline or somatic activating mutations in the *ALK* gene as well as amplification or rearrangements and/or over expression independently of its genetic status, providing evidence of its pivotal role in NB oncogenesis, growth and survival [3, 15, 27, 28]. Therefore, patients presenting with tumors exhibiting *ALK* aberrations, may potentially benefit from ALK-targeted therapy.

The use of ALK kinase inhibitors have been shown to be promising in treatment of Non-Small-Cell Lung Cancer (NSCLC), Anaplastic Large Lymphoma (ALL) and other cancers with an *ALK*-driven component, and for these reasons novel small-molecule inhibitors suitable for clinical applications are in development or in early-phase trials [29–31].

Furthermore, since *ALK* expression in normal adult tissues is found in very low levels, selective ALK inhibitors would exhibit sufficiently wide therapeutic windows in patients with *ALK*-activated cancers [31]. Thus, ALK represents a *bona fide* target and its inhibition is not predicted to result in undesirable systemic side effects [32].

Crizotinib, the first small-molecule kinase inhibitor, timely approved by Food and Drug Administration for the treatment of NSCLC patients (for details see FDA-US website), has limited activity against the various *ALK*-mutations identified in children with NB [14]. As observed with other kinase inhibitors, *ALK*-positive NSCLC patients eventually relapse on crizotinib. Several distinct mechanisms of crizotinib resistance have been identified but most of them as yet remain unknown and to be elucidated [33–37]. The crizotinib-resistance of NB cells with *ALK*^F1174L^ mutation should be surmountable with very high doses of the drug and/or with new generation of inhibitors [20]. Importantly, *ALK*^F1174L^ may secondarily arise as a mechanism of resistance after an initial response to crizotinib in patients with *ALK*-rearranged cancers [16].

Recently, Xcovery developed X-396, a second-generation highly specific ALK small molecule tyrosine kinase inhibitor. X-396 has proved to be more selective and up to 10 times more potent than crizotinib in NSCLC [10]. Preliminary clinical results showed that X-396 had efficacy in crizotinib resistant patients [19]. Accordingly, in the present study, X-396 proved to be more effective in inhibiting *in vitro* NB cell growth than crizotinib (IC_50_ 2.6-3.7 fold lower).

Importantly, we confirmed and extended the ability of X-396 to inhibit ALK phosphorylation in NB cell lines harbouring the two most common ALK mutations, F1174L and R1275Q [10]. Indeed, one of the most recent study undertaken in 1,596 diagnosed NB patients identified 8% mutations at three “hot spot” mutation sites that display the highest oncogenic potential in NB cells and account for 85% of mutations (R1275 43%, F1174 30%, and F1245 12%) [8], the most common of which, F1174L and R1275Q (73%), are present in some NB cell lines such as SH-SY5Y and LAN-5 that we have chosen for therapeutic approaches *in vivo* of the present study

Pharmacokinetic profiles performed in nude mice indicated that X-396 is slowly absorbed *in vivo* as indicated by T_MAX_ value of 2 h, followed by a moderate terminal plasma elimination half-life, thus indicate a favourable behaviour for clinical translation. The plasma and tumor concentrations were well above the IC_50_ required to inhibit the NB cell growth even at the low doses of 25 mg/kg or 50 mg/kg, but higher dose of 100 mg/kg is required to reach high enough concentration in the brain to achieve prolonged inhibition, if brain metastases were present. However, the determination of the brain concentration is mainly relevant from a toxicology perspective.

The development of acquired resistance to targeted therapies is considered a largely inevitable hurdle that has a substantial impact on patients [15, 38]. At least one mechanism of acquired resistance is the emergence of new *ALK* mutations at relapse of NB [27]. Therefore, alternative strategies that may overcome acquired resistance to therapeutic agents and increase the efficacy of small molecule inhibitors in *ALK*-mutated NB, are of particular relevance. One promising strategy could be the combination of kinase inhibitors with gene-specific silencing molecules, such as siRNAs, which are able to knockdown *ALK* independently of its mutational status by promoting degradation of the gene mRNA. In this context, we have recently generated targeted nanoliposomal formulations carrying *ALK*-directed siRNAs (TL[*ALK-*siRNA]), which are highly specific for the gene target [21, 22] and efficiently delivered to NB cells thanks to a selective tumor targeting provided by the carrier [24, 39].

Indeed, compared to free *ALK*-siRNA, TL[*ALK-*siRNA] formulations have low plasma clearance, increased siRNA stability, improved binding to NB cells, and are effective for *ALK*-silencing and induction of NB cell death [22]. Our liposomal formulation showed a strong *ALK* knockdown in mice-bearing NB tumors, which resulted in cell growth inhibition and prolonged survival, as described [21].

In the current study, we sought to combine the TL[*ALK-*siRNA] with the novel ALK-inhibitor X-396 showing, for the first time to our knowledge, that this drug combination exhibits a greater anti-tumor effectiveness in biologically and clinically relevant NB mouse models when compared with the single agents. Moreover, the combined treatment was also proven to be well tolerated, with no obvious toxicities (*i.e*. weight loss, skin rush), further underlining a potentially promising therapeutic translation.

In summary, this study provides solid evidence that a more sensitive and specific therapeutic approach for NB may be achieved by blocking the *ALK* signaling pathway simultaneously through the directed *ALK* gene knockdown by *ALK-*siRNA nanoliposomal formulation in combination with the pharmacological inhibition of the ALK kinase activity by X-396.

Although ALK mutations are found in a small percentage of neuroblastomas, other ALK activating aberrations may occur at genomic, or post-transcriptional or post-translational level (gene amplification, over expression, epigenetic regulation, phosphorylation). Therefore, the ALK role in the activation of downstream pathways involved in the tumorigenesis of NB might be far greater than expected.

We think that our data suggest that novel small molecule ALK inhibitors combined with RNA interfering-based nanoliposomes could ultimately subdue many ALK-driven cancers into manageable diseases.

## MATERIALS AND METHODS

### Reagent and chemicals

Lipids: Hydrogenated soy phosphatidylcholine (HSPC), cholesterol (CHE), 1, 2-distearoylsn-glycero-3-phosphoethanolamine-N-[methoxy (polyethylene glycol)-2000] (DSPE-PEG_2000_), 1, 2-distearoyl-sn-glycero-3-phosphoethanolamine-N-[maleimide(polyethylene glycol)-2000] (DSPE-PEG_2000_-MAL), 1, 2-dioleoy-1–3-trimethylamonium propane (DOTAP) were purchased form Avanti Polar Lipids, Inc. (Alabaster, AL). *ALK*-siRNA (siRNA ID# s1271) for liposome preparations was purchased from Ambion (Life Technologies, Carlsbad, CA).

Nuclepore polycarbonate membranes were purchased from Avestin Inc. (Ottawa, ON, Canada). 2-Iminothiolane (Traut's reagent) was obtained from Sigma-Aldrich (St. Louis, MO). Protein A/G column was purchased from Thermo Scientific-Pierce (Rockford, IL, USA). Bio-Rad Protein Assay Reagent was purchased from Bio-Rad Laboratories (Milano, Italy). Sephadex G-50, Sepharose CL-4B, aqueous counting scintillant and Cholesteryl-[1, 2-^3^H-(*N*)]hexadecyl-ether ([^3^H]CHE;1.48–2.22 TBq/mmol) was purchased from PerkinElmer Biosciences (Waltham, Ma, USA).

All other reagents were of analytical grade purity or the highest available purity and purchased from Sigma-Aldrich.

### Compounds

X-396 were provided by Xcovery (West Palm Beach, FL) through a materials transfer agreement, as a lyophilized white cake in 10 mL glass vials and stored at 4°C. Crizotinib were purchased from Selleckchem (Houston, TX 77054 USA; licensed by Pfizer). For the *in vitro* experiments, the appropriate amount of powder was dissolved in dimethyl sulfoxide (DMSO) to a final concentration of 10 mM and stored at −20°C until use. Reconstituted drugs were thawed and diluted in culture medium (to a final DMSO concentration less than 0.01%) immediately before use. The vehicle solution, obtained by mixing 0.5% Hydroxy Propyl-Methyl-Cellulose (HPMC) and 0.4% Tween-80 in 99.1% sterile water (w\w\v), was used to resuspend X-396 and crizotinib for the *in vivo* experiments.

### Cell lines and culture conditions

Two human Neuroblastoma (NB) cell lines carrying different *ALK*-mutations, SH-SY5Y (*ALK*^F1174L^) and LAN-5 (*ALK*^R1275Q^), were grown in Dulbecco's-modified-EM and RPMI 1640 medium (Sigma), respectively [21, 22]. Moreover, cells were tested for mycoplasma contamination, cell proliferation, morphology evaluation, and multiplex short tandem repeat profiling test, both after thawing and within six passages in culture.

### Cell proliferation and cell viability assay

NB cell lines were seeded in 96-well plates (at 1 × 10^3^ – 8 × 10^3^ cells per well) in complete medium and cultured for 24 hours. The medium was removed and replaced with fresh complete medium supplemented with four different concentrations of crizotinib or X-396 (10 – 2000 nM) or 0.01% DMSO (control). Six replicates of cells for each condition were then incubated for an additional 72 hours. Eighteen hours before the end of the treatment cells were then incubated with 0.5 μCi (0.0185 MBq) of ^3^H-thymidine (PerkinElmer, Waltham, Ma, USA) and processed for liquid scintillation counting (Packard Instruments Company, Downers Grove, IL) [40, 41].

For viability assay, two hours before the end of the treatment cells were incubated with AlamarBlue staining according to the manufacture's instructions (Life Technologies).

### Western blot analysis

NB cell lines were plated in 10 cm^2^-dishes and 24 hours after were treated with four different concentrations of crizotinib or X-396 (10 – 1000 nM) or 0.01% DMSO (Control) for in total 72 hours.

Total cell lysates were prepared and analyzed by western blot analysis [40, 42]. Briefly, cells lines were lysed with Cell Extraction Buffer (Life Technologies) *plus* protease-inhibitor cocktail (Sigma). Protein lysates (50 μg per lane) were resolved on sodium dodecyl sulphate (SDS) 8% polyacrylamide gels and transferred to nitro-cellulose membranes. Membranes were then incubated with antibodies against ALK and phospho-ALK tyrosine 1586 (Cat N°3333 and 3343 respectively, Cell Signaling Technology, Danvers, MA). Peroxidase-conjugated goat anti-mouse and anti-rabbit antibodies were used as secondary antibodies (Cell Signaling Technology). Immune complexes were visualized with the use of a Supersignal West Pico Chemiluminescent Substrate (Thermo-Scientific, Rockford, IL, USA) or Clarity Western ECL Substrate (Bio-Rad Laboratories, Milan, Italy) according to the manufacturer's instructions, and normalized to internal controls (a mouse monoclonal antibody against β-actin (Sigma)). Bands were quantified by using of Image J software (http://imagej.nih.gov/ij/index.html).

### Pharmacokinetic and therapeutic studies in subcutaneous NB model

Six-week-old male Balb/c nude mice (Beijing HFK Bio-Technology co. Ltd, Beijing, China) were subcutaneously injected with 4 × 10^6^ SH-SY5Y cells in matrigel. Once tumors reached an average volume of 250 mm^3^, 24 mice were randomly splitted in three groups and treated *via* oral gavage (OG) with either 25 or 50 mg/Kg of X-396 administered *bis in die* (BID) or 100 mg/kg of X-396 administered *quaque die* (QD) for 14 days. A uniform volume per administration (200 μL) was used for each group. Mice were monitored daily throughout the treatment period for sign of morbidity/mortality. On the last day of study, 0.1 – 0.2 mL of blood was collected from the X-396 treated mice as follows: 3 mice in each group were bled at pre-dose, 1, 4 hours post dose, and sacrificed at 4 hours.

Another 3 mice in each group were bled at 0.5, 2, and 8 hours post-dose, sacrificed at 8 hours. Tumor and brain tissues were explanted. Drug concentrations in plasma and tissues were analyzed by Liquid Chromatography-Mass Spectrometry (LC-MS)/MS [10].

In other set of experiments, Balb/c nude mice were xenografted with SH-SY5Y cells as earlier. Once tumors reached an average volume of 250 mm^3^, mice were randomized and treated as above or with either X-396 or crizotinib *via* OG, with 50 mg/Kg BID, or control vehicle. Tumors were measured twice weekly using callipers, and volume was calculated using the formula: Length × Width^2^ × 0.5. Body weight was also assessed twice weekly. The experiment was terminated after 3 weeks of treatment.

All studies were done in accordance with the current International Conference on Harmonization harmonized tripartite guidelines, and the protocol was approved by the Sundia Institutional Animal Care and Use Committee (Shangai, China).

### Preparation of Targeted Liposomes [*ALK-*siRNA]

Briefly, *ALK*-directed siRNA were complexed with cationic lipids and then coated with neutral lipids [22]. Successively, Fab' fragment of anti-GD_2_ monoclonal antibody was coupled to the malemide terminus of DSPE-PEG_2000_-MAL of the liposomes [24] to provide a formulation of Targeted Liposomes entrapping siRNA against *ALK* (TL[*ALK*-siRNA]). Liposomes size, polidispersity, and zeta-potential were analysed by dynamic light scattering using zeta-sizer Nano-S ZS90 particle sizer at a fixed angle (90°) (Malvern Instruments, Malvern UK).

### *In vivo* therapeutic studies in orthotopic and pseudometastic NB mouse models

All experiments involving these models have been reviewed and approved by the licensing and ethical committee of the IRCCS Azienda Ospedaliera Universitaria San Martino-IST Istituto Nazionale per la Ricerca sul Cancro, Genoa, Italy and by Italian Ministry of Health. All the *in vivo* experiments were performed using the least number possible in accordance with the 3 Rs policy.

In the orthotopic-model, NB cells (1.5 × 10^6^ LAN-5 or SH-SY5Y) were injected in the left adrenal gland capsule of five-week-old female athymic (*nu/nu*) mice (Harlan Italy, S. Pietro al Natisone, Udine, Italy) [23, 43]. No mice died as result of this treatment.

In the pseudometastatic model, 3 × 10^6^ SH-SY5Y cells were injected intravenously (*i.v*.) in the tail vein in four-week-old female *nu/nu* mice [24, 44]. Mice were then randomly assigned to the different groups and treated with either the vehicle alone (control mice) or with X-396 and/or TL[*ALK*-siRNA] (administered individually or in combination). Depending on the experimental schedules, mice received 50 or 100 mg/Kg of X-396 *via* oral gavage QD. An amount of 1 mg/Kg (20 μg/injection per mouse) of TL[*ALK*-siRNA] were *i.v*. injected twice a week, for a total of 3 weeks treatment, with a 3-day interval between injections.

Body weight and general physical status of the animals were recorded daily until they were judged to be in discomfort by animal caretakers. Specifically, once showing signs of poor health (*i.e*., abdominal dilatation, dehydration, paraplegia, severe weight loss), mice were euthanized by anaesthesia with xilezine (Xilor 2%, Bio98 Srl, Milan, Italy).

### Bioluminescent imaging for evaluation of primary therapeutic responses

A Bioluminescence Imaging System (BLI), able to capture signals released by Luciferase (Luc)-stably-transduced NB cells [45, 46], named SH-SY5Y-Luc and LAN-5-Luc, was used to monitor disease progression, metastasis and therapeutic effects in orthotopic-xenografted mice. Briefly, SH-SY5Y-Luc and LAN-5-Luc tumor cells, were orthotopically implanted into the left adrenal gland of *nu/nu* mice [45, 47]. The initial *in vivo* trafficking of the malignant cells, organ-specific homing, orthotopic expansion over time, and response to X-396 treatment were all readily visualized and quantified by a highly sensitive, cooled Charge-Coupled Device (CCD) camera mounted in a light-tight specimen box (IVIS Caliper Life Sciences, Hopkinton, MA) after 10 min incubation with 150 μg/mL of D-Luciferin (Caliper Life Sciences) [45].

Quantification of the *in vivo* Luciferase signal of the tumor area, expressed as fold increase of photon counts over time in the Region Of Interest (ROI), was computed at time 22 and 29 day with respect to the starting day of the treatment (T_0_).

### Statistical analysis

Results are expressed as mean values with 95% confidence intervals. All *in vitro* data are from at least three independent experiments. The statistical significance of differential findings between experimental and control groups was determined by Student's *t* test with Welch's correction or by one-way analysis of variance (ANOVA) with the Tukey's or with the Bonferroni's multiple comparison test. IC_50_ values were evaluated by non-linear regression (curve-fit: log inhibitor *vs* normalized response-variable slope) and significance between slopes were evaluated by linear regression, Best-fit values and Spearman r (two-tailed) in GraphPad Prism 5.03 software (GraphPad Software, Inc., La Jolla, CA). These findings were considered significant if two-tailed *P* values were < 0.05.

All *in vivo* experiments were repeated at least two times with similar results. Kaplan-Meier method & Log Rank (Mantel-Cox) test was applied to build up and compare the survival curves between analyzed groups.

## SUPPLEMENTARY FIGURES


