# Supplementary material for: New therapeutic strategies in neuroblastoma: combined targeting of a novel tyrosine kinase inhibitor and liposomal siRNAs against ALK
Source: Oncotarget. 2015 Jun 20;6(30):28774–89. doi: 10.18632/oncotarget.4342 (PMC4745691; doi:10.18632/oncotarget.4342)
Supplement: Supplementary file 1 [file oncotarget-06-28774-s001.pdf]

SUPPLEMENTARY FIGURES

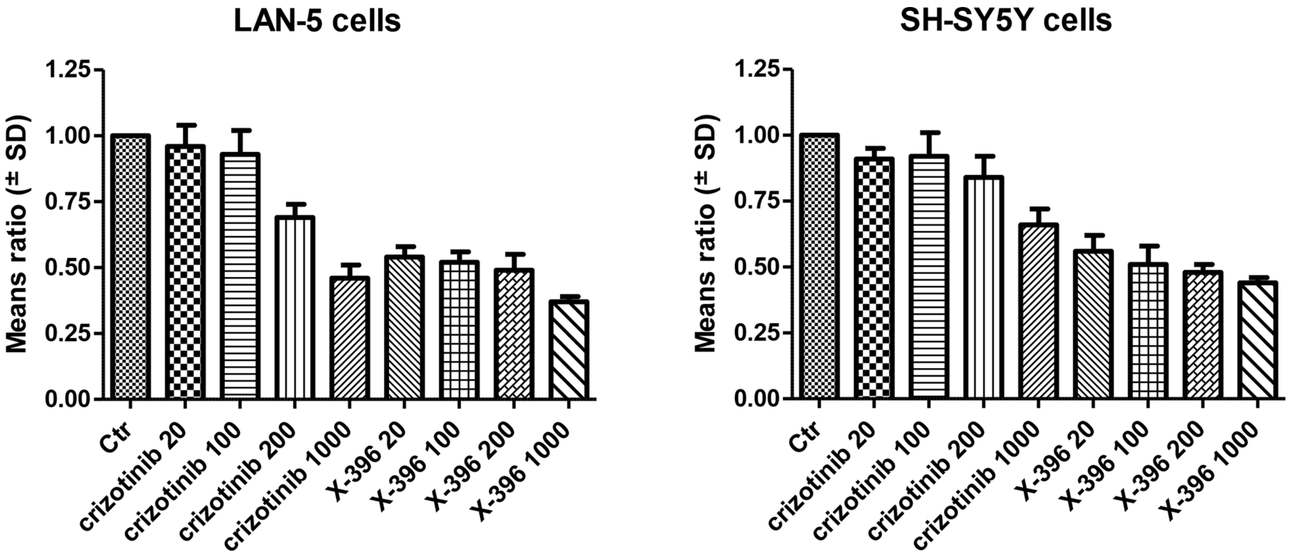

Supplementary Figures S1: Quantification of the western blot bands presented in Figure 1E, 1F, are here expressed as means of the ratio between treated cells and controls, after β-actin normalization, of three independent experiments.

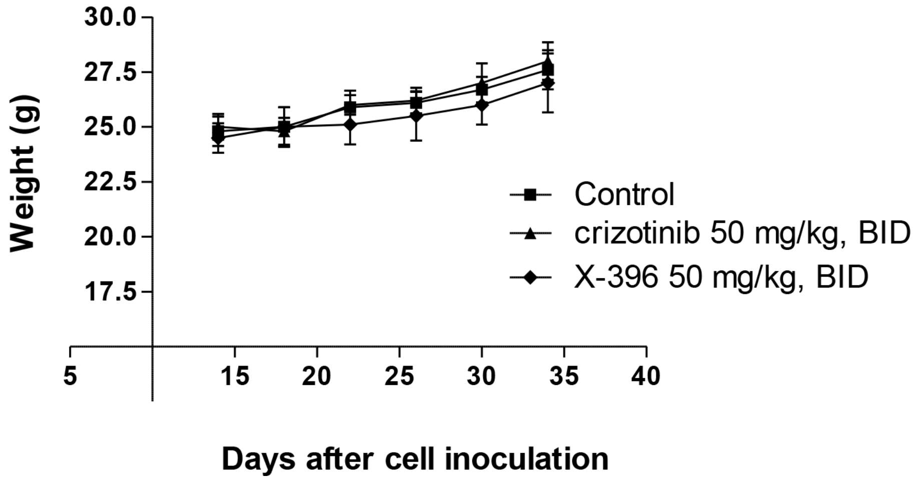

Supplementary Figure S2: Comparison of X-396 and crizotinib administered at the same dose. NB-bearing mice were OG treated with 50 mg/kg BID of X-396 or crizotinib and mice weight determined over time. Error bars ± SD. No signs of severe weight loss were observed during the treatment.
